# Daidzein improves muscle atrophy caused by lovastatin by regulating the AMPK/FOXO3a axis

**DOI:** 10.1186/s13020-024-01034-5

**Published:** 2024-12-31

**Authors:** Keke Wang, Hao Zeng, Hua Yang

**Affiliations:** State Key Laboratory of Natural Medicines, School of Traditional Chinese Pharmacy, China Pharmaceutical University, Nanjing, 210009 China

**Keywords:** Lovastatin, Skeletal muscle atrophy, Daidzein, AMPK, FOXO3a, Zebrafish

## Abstract

**Background:**

Lovastatin, the main lipid-lowering component in red yeast rice, is a golden anti-lipid drug, but its long-term application is continuously challenged by potential skeletal muscle atrophy. Daidzein, an isoflavone derived from soybeans and many Chinese medicines, shows therapeutic potential in treating muscle-related diseases and metabolic disorders. However, whether daidzein can improve lovastatin-induced muscle atrophy and the specific mechanism needs to further study.

**Methods:**

Lovastatin-induced mice and zebrafish muscle atrophy models were used to validate the protective effect of daidzein in vivo. And the lovastatin-induced C2C12 myotube atrophy model was employed to validate the therapeutic efficacy and investigate the specific mechanism of daidzein in vitro. We combined specific siRNA targeting FOXO3a and AMPK-selective inhibitor, agonist to elucidate AMPK/FOXO3a-dependent muscle-protective mechanism of daidzein. The anti-atrophy effects of daidzein through blockage of abnormal activation of AMPK/FOXO3a was presented in Immunofluorescence, H&E staining, Western blot, qRT-PCR. Serum creatine kinase level was detected by ELISA and we used mouse muscle grip instrument to detect the strength of mouse muscles.

**Results:**

In this study, we demonstrated that daidzein could dose-dependently alleviate lovastatin-induced mice skeletal muscle atrophy, reduce serum creatine kinase, and improve muscle grip strength in mice. Mechanistically, daidzein inhibited lovastatin-induced FOXO3a phosphorylation caused by AMPK activation, thereby inhibiting FOXO3a nuclear translocation to restrain the expression of muscle-related proteins Atrogin-1 and MuRF-1. In C2C12 myotube, administration of AMPK-selective inhibitor Compound C recapitulated the therapeutic effects of daidzein against lovastatin-induced myotubes atrophy, while the anti-atrophy effects of daidzein were lost in the presence of AMPK-selective agonist MK-3903. In lovastatin-induced mice muscle atrophy models, Compound C elicited similar anti-atrophy effects as daidzein, but this effect was not potentiated when it was applied in combination with daidzein, suggesting that daidzein exerted therapeutic efficacy dependent on blockage of AMPK activity.

**Conclusions:**

Our study identified daidzein as an effective component that ameliorated lovastatin-induced skeletal muscle atrophy through blockage of abnormal activation of AMPK/FOXO3a and transcriptional activation of genes encoding downstream muscle-related proteins. Our results also highlighted the therapeutic potential by regulating the AMPK/FOXO3a axis in management of statin-induced myotoxicity.

**Supplementary Information:**

The online version contains supplementary material available at 10.1186/s13020-024-01034-5.

## Background

Statins, also known as HMG-CoA reductase inhibitors, are primarily used to lower serum cholesterol levels, including total cholesterol and LDL cholesterol. Meanwhile, administration appropriate amounts of statins can not only lower morbidity from coronary heart disease but also prevent stroke [1–3]. In general, statins are well tolerated but may cause potentially adverse effects, particularly in skeletal muscle. For example, myopathy has occurred in 1.5–5% of patients taken by statins, where rhabdomyolysis is even rarer [4]. The potential muscle toxicity characterized by muscle atrophy caused by statins, which is involved in reducing muscle mass and weakening muscle grip strength. The muscle protein degradation and synthesis are closely related to muscle regulation, and both food deprivation and some major disease types (such as: uremia diabetes mellitus, sepsis, heart failure or cancer cachexia) can rapid lower the muscle weight [5, 6]. Under these situations, muscle proteins are quickly regulated by a mechanism relative to identical transcriptional and biochemical regulators, including the activation of the ubiquitin–proteasome system, which is an important intracellular system for protein degradation [7, 8]. Atrogin-1 and MuRF-1(ubiquitin-protein ligase or E3) play a key role in atrophying muscle [9, 10]. Atrogin-1 and MuRF-1 are induced in the early stage of atrophying muscle process, and the increasing in Atrogin-1 and MuRF-1 expression occurs before the muscle weight loss [9]. It has been reported that Atrogin-1 and MuRF-1 play a key role in statin-induced atrophying muscle [11–13]. FOXO3a, as the major transcription factor of MuRF-1 and Atrogin-1, is closely related to the development of atrophying muscle [14–16]. Studies have reported that statins can inhibit the phosphorylation of FOXO3a and inhibit its nuclear transfer [17–19]. However, the specific mechanism of FOXO3a and statin-induced muscle atrophy is still unclear.

AMPK inhibits many anabolic processes, enhances protein degradation and autophagy [20, 21], and lowers blood glucose [22, 23]. Therefore, AMPK plays a vital function in controlling the skeletal muscle growth and development [20]. An AMPK agonist Metformin is a classic hypoglycemic drug by inhibiting glucose absorption, but its long-term application is continuously challenged by potential skeletal muscle atrophy [24–26]. The study has shown that simvastatin can cause mitochondrial damage in rat cardiomyocytes, leading to FOXO3a phosphorylation downstream AMPK activation, thereby inhibiting FOXO3a nuclear translocation [12]. Statins can activate AMPK mainly through two mechanisms. On the one hand, AMPK, as an energy receptor, can sense the change of intracellular cholesterol and statin activates AMPK by reducing the content of intracellular cholesterol. On the other hand, statins can increase the phosphorylation level of AMPK at threonine 172 by enhancing the activation of Rac1 [27]. Besides, the clinical data showes that the phosphorylated form of AMPK is increased in statin-treated patients and human muscle biopsies from four patients with muscle symptoms related to statin therapy showe ATP content reduction which indicates the involvement of AMPK [28, 29]. Therefore, AMPK is closely related to statin-induced muscle atrophy.

Daidzein (C_15_H_10_O_4_), an isoflavone with a structure similar to estrogen and various pharmacological activities, is extracted from soybeans and many Chinese medicines (eg. red yeast rice or pueraria lobate) [30]. For example, daidzein can combine with α and β estrogen receptors to show the effects of estrogen on their target organs [31, 32]. Daidzein possesses a protective action on metabolic disorders such as hyperlipidemia and diabetes [33]. Besides, daidzein has potential in treating muscle-related diseases. For example, the clinical data shows supplementation with soy isoflavones which the main ingredient is daidzein can increase lean mass and muscle mass index (assessment of skeletal muscle mass in humans) in obese-sarcopenic postmenopausal women [34]. And it has been reported that daidzein decreases the expression of ubiquitin-specific protease 19 and improves skeletal muscle mass through estrogen receptorβ [35]. Daidzein can ameliorate skeletal muscle atrophy induced by chemotherapy drug cisplatin [36]. Supplementing a certain amount of isoflavones in diet can also alleviate muscle atrophy caused by tumor load in mice [37]. However, whether daidzein can improve lovastatin-induced muscle atrophy needs further study.

Our study found that in C2C12 myotubes, daidzein inhibited lovastatin-induced FOXO3a phosphorylation downstream AMPK activation, thereby lowering FOXO3a nuclear translocation to restrain expression of muscle-related proteins Atrogin-1, MuRF-1. Knockdown of FOXO3a reversed lovastatin-induced myotube atrophy, which demonstrated that lovastatin induced myotube atrophy in a FOXO3a-dependent manner. However, adding AMPK-selective agonist MK-3903 abolished the effect of daidzein. In zebrafish embryos, daidzein ameliorated the skeletal muscles damages and decreased muscle capacity caused by lovastatin. Intragastric administration of daidzein (100 and 150 mg/kg/day) to mice ameliorated lovastatin-induced muscle atrophy, decreased the serum creatine kinase, and enhanced the muscle grip strength in mice. Administration of AMPK-selective inhibitor Compound C recapitulated the therapeutic effects of daidzein against lovastatin-induced myotubes and mice atrophy. These results indicated that daidzein exerted therapeutic efficacy on lovastatin-induced muscle atrophy through restricting AMPK/FOXO3a pathway. At present, there are no applicable preventive methods and drugs to manage the muscle atrophy caused by statins. Therefore, our study identified daidzein as an effective component that ameliorated lovastatin-induced skeletal muscle atrophy through blockage of abnormal activation of AMPK/FOXO3a and transcription of genes encoding downstream muscle-related proteins. Our study also highlighted the therapeutic potential by pharmacological targeting AMPK/FOXO3a axis in the management of statin-induced myotoxicity.

## Materials and methods

### Cell culture and main reagents used

The C2C12 cell line was obtained from Shanghai Cell Bank, Chinese Academy of Sciences and the C2C12 cell line was cultured in medium (DMEM, KeyGEN BioTECH, China) with 10% fetal bovine serum (FBS, Gibco, USA). Lovastatin (MCE, HY-N0504), Daidzein (Yuanye, 486–66-8), MK-3903(MCE, HY-107988), Compound C (Yuanye).

### Differentiation of C2C12 myotubes

The C2C12 cells are seeded into cell culture dishes. When the cell density reaches 90%-95%, the medium containing horse serum (Gibco, USA) is replaced to induce cell differentiation. The induction lasted for 6–8 days with daily medium changes. Successfully differentiated myotube cells are formed by the fusion of multiple cells and exhibit a multinucleated morphology.

### Animal experiments

Care and surgical methods for the mice were conducted following the Guide for the Care and Use of Laboratory Animals by the National Institutes of Health and approved by the Animal Care Committee of China Pharmaceutical University. In order to study whether daidzein could alleviate lovastatin-induced muscle atrophy, 6–8 weeks old male C57BL/6 J mice from Laboratory Animal Center of Hangzhou Ziyuan (Hangzhou, China) were divided into the following groups. The Control group (CON) was treated with 0.5% CMC-Na solution (n = 8). Lovastatin group was treated with lovastatin (LV, 80 mg/kg/day) (n = 8). Daidzein group was treated with daidzein (DA) (150 mg/kg/day) (n = 8). Lovastatin + Daidzein group was treated with lovastatin (80 mg/kg/day) plus daidzein 50 mg/kg/day (L), 100 mg/kg/day (M), 150 mg/kg/day (H) (n = 8). After 2 months of continuous intragastric administration, the mice were anesthetized and their blood and gastrocnemius muscles were collected.

To prove that daidzein relieved lovastatin-caused muscle atrophy by blockage the abnormal activation of AMPK, 6-8 weeks old male C57BL/6J mice from Laboratory Animal Center of Hangzhou Ziyuan (Hangzhou, China) were divided evenly into five groups. The Control group was treated with 0.5% CMC-Na solution (n=6). Lovastatin group was treated with lovastatin (80 mg/kg/day) (n=6). Lovastatin + Daidzein group was treated with lovastatin (80 mg/kg/day) and daidzein (150 mg/kg/day) (n=6). Lovastatin + Daidzein + Compound C group was treated with lovastatin (80 mg/kg/day) and daidzein (150 mg/kg/day) and Compound C (20 mg/kg/day) (n=6). Lovastatin + Compound C group was treated with lovastatin (80 mg/kg/day) and Compound C (20 mg/kg/day) (n=6). After 2 months of continuous intragastric administration, all of the mice were anesthetized and their blood and gastrocnemius muscles were collected.

### Mouse muscle grip strength test

We used grip Strength Meter (YLS-13A, Shandong) to detect the grip strength of mice limbs. Label each mouse and place it flat on the mouse muscle grip strength testing instrument. Use our hand to lift the mouse's tail, stabilize the mouse's forelimbs, and pull the mouse with the same force. Repeat this process 6 times, then take the average of the 6 values obtained as the maximum muscle grip strength of the mouse.

### Immunofluorescence staining of FOXO3a and MyHC

The cells were seeded on a confocal dish. After the cells were completely attached, administrated with drugs for 24 h. Discard the drug-containing medium, rinse the cell culture with PBS three times for 5 min each time, and fix with 4% paraformaldehyde for 15 min. Rinse the cell culture with PBS once, then permeabilize with 0.5% Triton X-100 (Sigma-Aldrich, T8787) for 10 min. Incubate with 3% BSA for 1.5 h at 37 °C, then incubated with FOXO3a antibody (proteintech, 10,849-1-AP) overnight at 4 °C. Then, we washed the cells three times with cellular chamber PBS, added secondary antibody and incubated at 37 °C. Finally, we washed with cellular chamber PBS three times, added DAPI and incubated at 37 °C for 10 min. The confocal microscopy was used to capture the image.

C2C12 cell were seeded on the 12 well plate. When the cell density reached 90–95%, the medium containing horse serum was replaced to induce cell differentiation for 6–8 days. Then, based on different groups, different drugs were administered for 24 h. Discard the drug-containing medium, rinse the cell culture with PBS three times for 5 min each time, and fix with 4% paraformaldehyde for 15 min. Rinse the cell culture with PBS once, then permeabilize with 0.5% Triton X-100 (Sigma-Aldrich, T8787) for 10 min, incubated with 5% BSA for 1.5 h at 37 °C, then incubated with MyHC (RD, MAB4470) overnight at 4 °C. Finally, we washed the cells three times with cellular chamber PBS, added secondary antibody and incubated at 37 °C for 2 h, washed with cellular chamber PBS three times.

### Detection of the creatine kinase activity in mice serum

Allow the collected mouse blood to stand at room temperature for 4 h, then centrifuge at 3000 rpm for 10 min. Aspirate the mouse serum and store it at − 80 °C. Take the mouse serum out of the − 80 refrigerator and place it on ice. We used the mouse Creatine kinase kit (Solarbio, BC1145) to detect the activity of creatine kinase in mouse serum and detected the samples using an enzyme-linked immunosorbent assay.

### Cytoplasmic and nuclear extraction

We used a cytoplasmic and nuclear extraction kit (Beyotime, P0027) to isolate the nucleus and cytoplasm of mouse gastrocnemius muscle tissue and C2C12 myotubes. Weigh a certain amount of gastrocnemius muscle, add an appropriate volume of cytoplasmic lysis buffer, and add magnetic beads. After grinding with a tissue homogenizer, proceed with the extraction according to the requirements of the kit. Rinse the cell samples twice with cell culture PBS, then add trypsin for cell digestion and collection according to the requirements of the kit.

### Western blot

We used RIPA (Beyotime, P0013C) and to collect the total protein from cells and mouse gastrocnemius muscle. Weigh the gastrocnemius muscle tissue from adapted mass mice. Add an appropriate volume of lysis buffer, 10 times the muscle mass, and add grinding beads. Grind with a tissue homogenizer, then incubate on ice for 1 h, followed by centrifugation at 12,000 rpm for 15 min. Collect the supernatant, perform BSA protein quantification, and based on the protein quantification results, add the appropriate volume of loading buffer and mix well. Boil at 100 °C Celsius for 10 min and store at − 80 °C Celsius for later use. Rinse the cell samples with cell culture PBS three times. Add an appropriate volume of lysis buffer based on the cell amount, incubate on ice for 10 min, then, centrifuge at 12,000 rpm for 15 min. Collect the supernatant, add the appropriate volume of loading buffer based on the volume of the supernatant, mix well, and boil at 100 °C Celsius for 10 min. Immunoblotting was used to test the proteins expression according to a standard method. After SDS-PAGE gel electrophoresis according to the groups, transfer the proteins from the gel onto NC membranes. Incubate the membrane at room temperature with 3% skim milk powder for 2 h, then shake with the primary antibodies overnight at 4 °C Celsius. After rinsing with TBST, incubate the protein on the NC membrane with the secondary antibodies for 1.5 h at room temperature. After TBST washing, visualize the NC membrane with the ECL chemiluminescence reagent using a Tanon imaging system. PCNA, FOXO3a, Atrogin-1/MAFbx, MuRF-1 were obtained from Proteintech (Wuhan). AMPK/p-AMPK primary antibodies were purchased from Affinty Biosciences (Jiangsu). The secondary antibodies were purchased from Beyotime Biotechnology (Shanghai). F59 primary antibody (AB_-_528373) was purchased from Developmental Studies Hybridoma Bank. MYHC primary antibody (MAB4470) was purchased from R&D system. The p-FOXO3a (ET1609-49) was purchased from HUABIO (Hangzhou).

### Quantitative real-time PCR

We used Trizol (Vazyme, R401–01) to collect the RNA of cells, mouse gastrocnemius muscle and zebrafish embryo. Detect the concentration of RNA from different groups, and reverse transcribe the corresponding amount of RNA into cDNA according to the RNA concentrations. Real-time PCR were used to test the mRNA expression of *Fbxo32* and *Murf-1*.The mouse target gene primers are showed as follows: *Fbxo32* (Forward 5ʹ—CTT CTC GAC TGC CAT CCT GGA T-3’); (Reverse 5ʹ—TCT TTT GGG CGA TGC CAC TCA G-3’); *Murf-1* (Forward 5ʹ—TAC CAA GCC TGT GGT CAT CCT G-3’); (Reverse 5ʹ—ACG GAA ACG ACC TCC AGA CAT G-3’); *Gapdh* (Forward 5ʹ—ATG CCA GTG AGC TTC CCG TTC AG—3ʹ); (Reverse 5’-CAT CAC TGC CAC CCA GAA GACTG—3ʹ). The expression levels of mRNA was calculated using *Gapdh* as an internal control and represented as mean ± standard error of mean (SEM). The zebrafish target gene primers are showed as follows: *Fbxo32* (Forward 5ʹ—AAG CTC TGC CAG TAT CAC TTC—3ʹ); (Reverse 5ʹ— AGT GCA AGG ATG GTC TGT ATC—3ʹ); *Murf-1* (Forward 5ʹ—GAC AAT GCT CAA CGT GCC AA—3ʹ); (Reverse 5’- AAC GCC TAG CCA AAC TCC TG—3ʹ); *rpl13a* (Forward 5ʹ—CGC TAT TGT GGC CAA GCA AG—3ʹ); (Reverse 5ʹ—TCT TGC GGA GGA AAG CCA AA—3ʹ). The expression levels of mRNA was calculated using *rpl13a* as an internal control and represented as mean ± standard error of mean (SEM).

### Myotube measurement and analysis

We used Nikon inverted microscope to capture the C2C12 myotubes images. For each well, diameters of more than 150 myotubes were detected and we used ImageJ to measure the diameter.

### Cell transfection

The siRNA FOXO3a and AMPK was purchased from Gene Pharma Co., Ltd. (Shanghai, China). C2C12 cells were seeded on the 6 well plate. When the cell density reached 90%-95%, the medium containing horse serum was replaced to induce cell differentiation for 6–8 days. When cell differentiation was complete, the myotubes were transfected with siRNA FOXO3a and AMPK using transfection reagent Lipofectamine 3000 (Invitrogen, L3000015) according to the protocol. After 48 h transfection, we collected the protein and checked the transfection efficiency and proceed with the next experiment.

### Hematoxylin and eosin staining

After euthanizing the mice, use surgical scissors to obtain the gastrocnemius muscle. Weigh the muscle and then place it into 4% paraformaldehyde for fixation for 48 h. After fixation, dehydrate the tissue and embed it in paraffin wax. Then, slice the muscle tissue horizontally into 4 µm sections. After dewaxing the paraffin sections, stain them with a solution of hematoxylin and eosin (G1121, Solarbio).

### Breeding and propagation of zebrafish

The optimal growth temperature for zebrafish is 28.5 °C, with a pH range of 7.0–8.0. They require a light environment for 14 h per day and darkness for 10 h per day. Feed them twice daily with enriched brine shrimp and change the water in the fish tank regularly. For zebrafish breeding, two male and two female fish were placed into a breeding tank the night before last. A divider was used to separate the males and females. The water temperature was maintained at an appropriate level, and the tank was kept in darkness until the start of the light cycle the next morning. The divider was removed at the beginning of the light cycle. After 15 min, the females and males began mating. After mating for 2 h, the fish eggs could be collected. Place the eggs in zebrafish embryo culture medium and place the culture dish in an incubator. In the evening, remove any dead or unfertilized embryos.

### Staining of zebrafish embryo muscle tissue

Add 0.003% PTU (1-phenyl-2-thiourea) to the fertilized zebrafish embryo culture medium. After incubating the embryos in the incubator for 48 h, use a pipette to transfer the hatched larvae into separate wells of a 6-well plate. Administer different drugs according to the respective groups for 24 h. Wash the zebrafish embryos three times with embryo culture medium, then fix them in 4% paraformaldehyde solution at 4 °C for 10 h. After rinsing with embryo culture medium, immerse the zebrafish in methanol at − 20 °C for 2 h, followed by permeabilization with acetone for 30 min. Rinse three times with PBS, block with 3% BSA for 2 h, then, rinse three times with PBS. Incubate with zebrafish MyHC antibody overnight at 4 °C, followed by three rinses with PBS. Incubate with fluorescent secondary antibody at room temperature for 1.5 h, then rinse with PBS. Mount the zebrafish on glass slides using glycerol and capture images under a confocal microscope.

### Zebrafish locomotion capability testing

Transfer 48 h-old zebrafish larvae into a 6-well plate using a pipette for drug treatment. After 24 h of treatment, place one fish per group into each well of a 24-well plate and add an appropriate amount of embryo culture medium. Insert the 24-well plate into a zebrafish behavioral analysis instrument, adjusting the interval vibration mode. Zebrafish exhibit sudden movements in response to stimuli. Based on the speed of zebrafish movement, categorize them into three types: red for fast movement, green for moderate movement, and no movement. This method allows for the assessment of zebrafish locomotion capability. The red and green lines in the graph represent the trajectories and speeds of zebrafish movement, reflecting their locomotion ability.

### Cell viability testing

The C2C12 cells were seeded on the 96 well plate. When the cell density reached 90–95%, the medium containing horse serum was replaced to induce cell differentiation. The induction lasted for 6–8 days with daily medium changes. When the myotubes have successfully differentiated, simultaneously administer 10 µmol/L of lovastatin and gradient concentrations (12.5, 25, 50, 100 µmol/L) of daidzein for 24 h. Discard the culture medium containing the drugs. Rinse twice with PBS and add 100 µl of culture medium containing 10% CCK-8 per well. Incubate the plate in a 37 °C cell incubator for 30 min, then measure the absorbance at 450 nm using a microplate reader.

### Molecular docking

Autodock was used to predict the binding of daidzein to AMPK. Found the protein structures of several subunits of AMPK in the RCSB Protein Data Bank.

### Statistical analysis

Statistical analysis was conducted using GraphPad Prism 8.0.1 software. Experimental data are presented as ± SEM, with p < 0.05 indicating significant differences, p < 0.01 indicating highly significant differences, and p < 0.001 indicating extremely significant differences. If the obtained data conform to normal distribution and homogeneity of variance, one-way analysis of variance (One-way ANOVA) was used for multiple group comparisons, and pairwise comparisons were performed using the LSD test. If the data did not meet the assumption of equal variance, Dunnett’s T3 test was employed.

## Results

### Lovastatin can cause C2C12 myotubes atrophy and upregulate the expression of muscle-related proteins

**Fig. 1:**
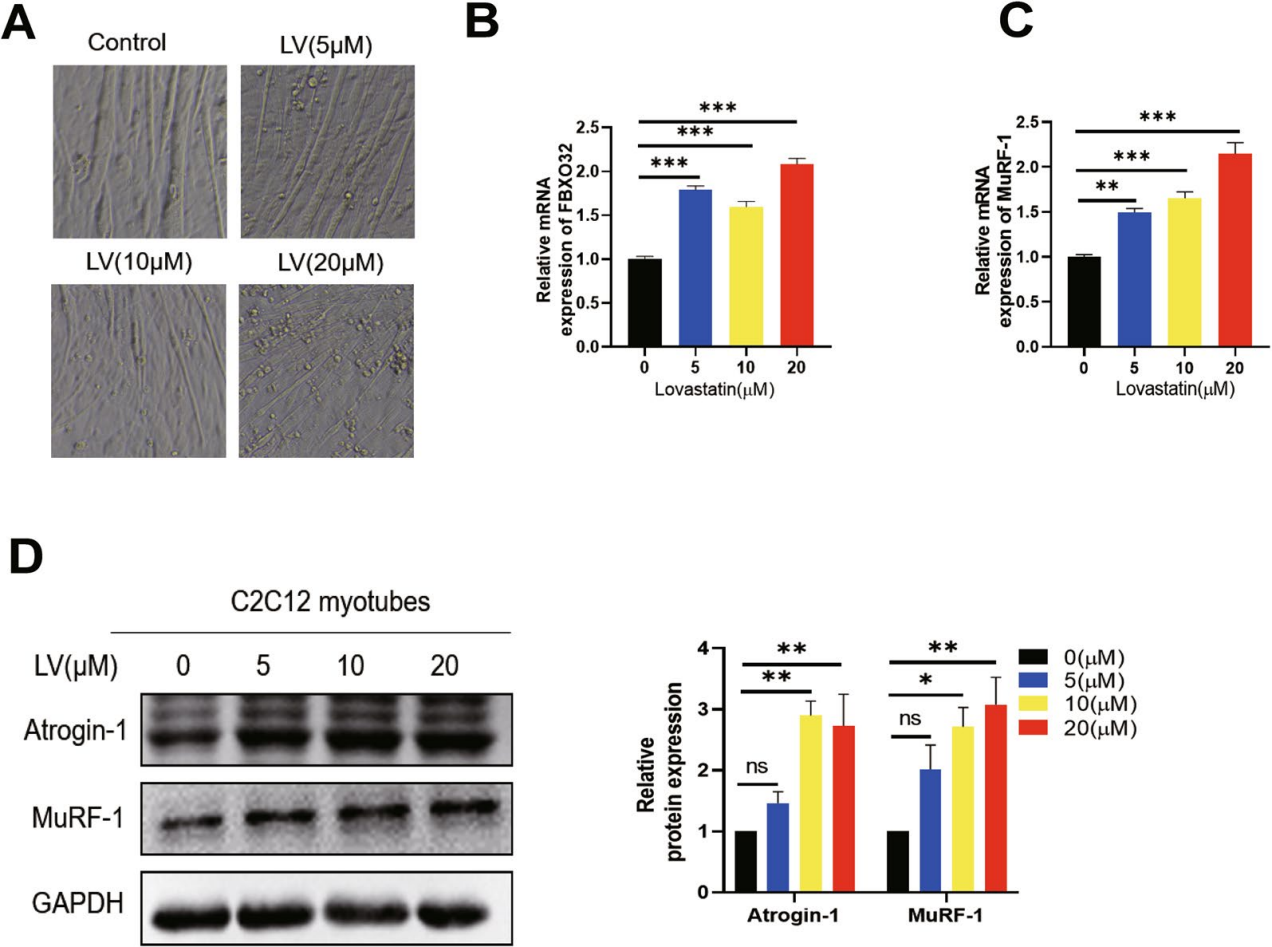
Lovastatin can cause C2C12 myotubes atrophy and upregulate the muscle-related proteins level.** A** C2C12 myotubes were administered with lovastatin (5 µmol/L, 10 µmol/L and 20 µmol/L) for 24 h and the inverted microscope was used to capture the pictures. **B-C** C2C12 myotubes were administered with (LV) lovastatin for 24 h, total cell RNA was extracted, and RT-qPCR was used to test the RNA level of *Fbxo32* and *Murf-1*. **D** C2C12 myotubes were administered with lovastatin for 24 h, cellular proteins were extracted; immunoblotting was used to test the expression of Atrogin-1 and MuRF-1. The image J was used to quantify the proteins level. The mean ± SEM was used to present the data. *p < 0.05; **p < 0.01; ***p < 0.001, (n = 3)

Lovastatin is a first-generation statin that commonly used in clinical setting, and it is widely applied to study statin-induced muscle toxicity in multiple model organisms and cellular models including mice, zebrafish and C2C12 myotubes, etc. [13, 38–40]. Therefore, we chose lovastatin in this study. In order to study the effect of lovastatin on skeletal muscle cells, we administered different concentrations of LV (lovastatin) to C2C12 myotubes for 24 h. Compared with Control group, after administration of lovastatin, the diameter of C2C12 myotubes decreased with increasing lovastatin concentrations (Fig. 1A). The mRNA and protein levels of muscle degradation-related proteins Atrogin-1 and MuRF-1 were dose-dependently increasing with lovastatin concentrations (Fig. 1B–D). According to the reference [13] and our experiment results, we selected 10 µmol/L lovastatin as the modeling concentration for statin-induced C2C12 myotubes atrophy.

### Daidzein reverses lovastatin-induced C2C12 myotubes atrophy

**Fig. 2:**
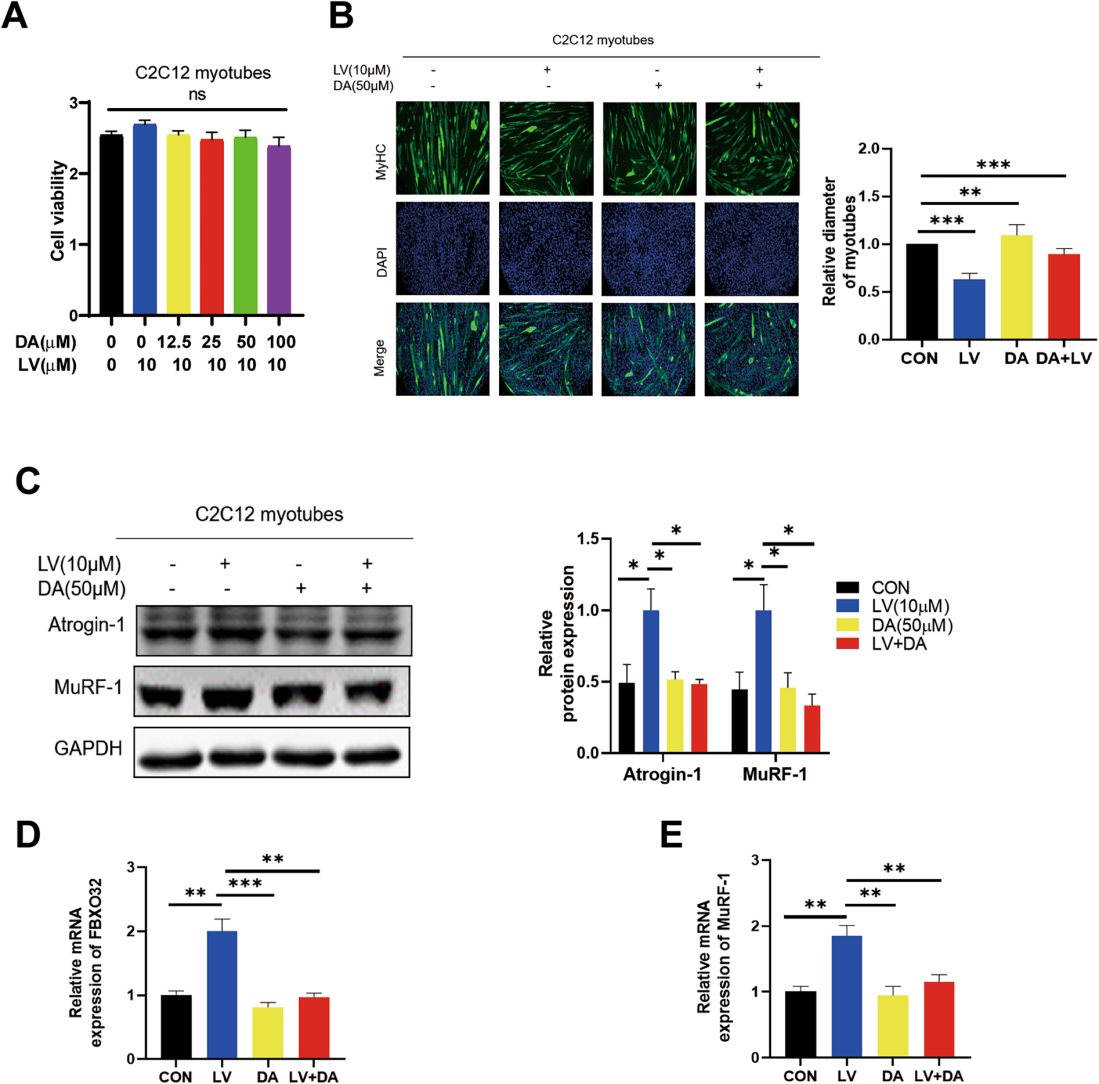
(DA) daidzein reverses (LV) lovastatin-induced C2C12 myotubes atrophy.** A** C2C12 myotubes were administered with 10 µmol/L lovastatin and different concentration of daidzein for 24 h and incubated CCK-8 for 1.5 h, and tested with Microplate reader(n = 6). **B** C2C12 myotubes were administered with 10 µmol/L lovastatin for 24 h, MyHC was stained, and inverted microscope were used to capture the images. C2C12 myotubes were calculated and analyzed with ImageJ. **C**–**E** C2C12 myotubes were treated with lovastatin + 50 µmol/L daidzein for 24 h and cellular protein and RNA were collected. Immunoblotting and RT-qPCR were used to test the expression level of Atrogin-1 and MuRF-1. The mean ± SEM was used to represent the data. *p < 0.05; **p < 0.01; ***p < 0.001, (n = 3)

Under 24 h intervention conditions, we found that DA (daidzein) had no significant effect on C2C12 myotubes and C2C12 cells viability (Fig. 2A, Fig S2 A). Besides, the results showed that daidzein alone at 50 µmol/L had no significant effect on the intracellular ATP content, thus excluding the potential cytotoxicity. We found that daidzein at 50 µmol/L could significantly reverse the lovastatin-induced reduction in C2C12 myotubes diameters (Fig. 2B). At the same time, compared with LV group, we found that daidzein reversed the upregulation of muscle-related proteins Atrogin-1 and MuRF-1 protein and mRNA levels caused by lovastatin (Fig. 2C–E). Zebrafish has fast reproduction, low cost and small size, and the whole fish body is muscle, which is a good model for studying muscle injury [13]. The study has shown that that lovastatin can result in muscle damages and destroy muscle fibers in zebrafish larvae. We then further verified the efficacy of daidzein in zebrafish. 48 h zebrafish embryos were given lovastatin and daidzein at the same time for 24 h. Compared with CON (control group), daidzein reversed the lovastatin-induced zebrafish muscle fibers damage, improved zebrafish’s locomotor ability and reversed the upregulation of muscle-related proteins Atrogin-1 and MuRF-1 protein and mRNA levels caused by lovastatin (Fig. S1 A, B, Fig. S4 A, B), which further proved that daidzein could reverse lovastatin-induced muscle atrophy.

### Daidzein improves lovastatin-induced skeletal muscle atrophy in mice

**Fig. 3:**
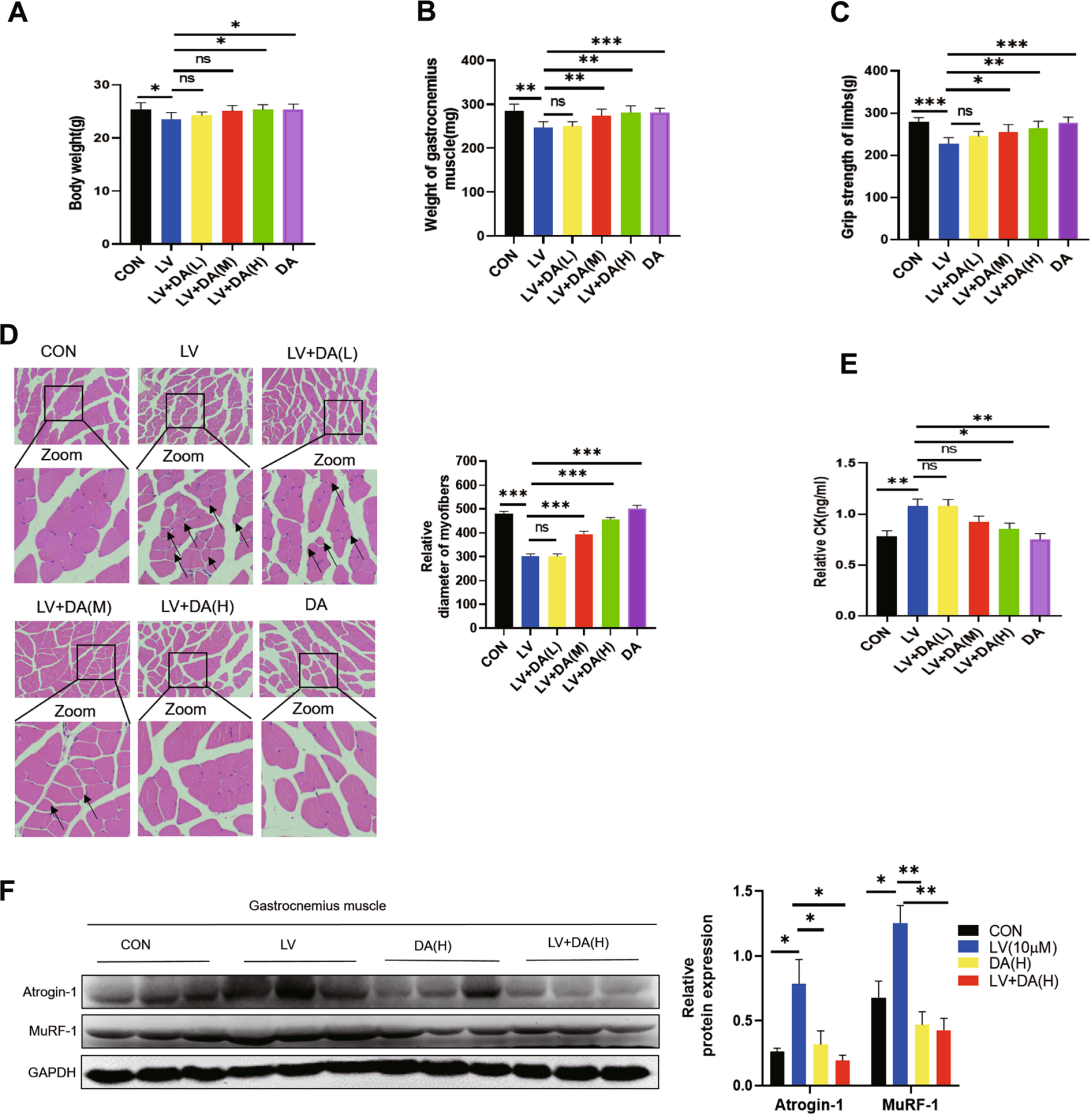
Daidzein alleviates lovastatin-induced skeletal muscle atrophy in mice. The mice were evenly divided as follow (n = 8). After treatment with LV (lovastatin, 80 mg/kg/day), DA (daidzein, 150 mg/kg/day), and LV (lovastatin, 80 mg/kg/day) combined with DA (L, M, H)-(daidzein, 50,100,150 mg/kg/day) for 2 months, the mice were detected. **A** The mice body weight.** B** Weight of gastrocnemius muscle. **C** Grip strength. **D** Hematoxylin–eosin staining of mice gastrocnemius muscle (scale bar = 50 µm). **E** Serum CK activity. **F** Extract protein from gastrocnemius muscle of mice in each group and immunoblotting was used to test the protein expression levels. The image J was used to quantify the protein level. The mean ± SEM was used to present the data. *p < 0.05; **p < 0.01; ***p < 0.001, (n = 3)

We have demonstrated that daidzein could improve reverse lovastatin-induced muscle atrophy in C2C12 myotubes and zebrafish models. In order to verify the efficacy of daidzein, we selected the mouse model of skeletal muscle atrophy by lovastatin [11, 39, 40], and gave daidzein (50, 100, 150 mg/kg/day by gavage simultaneously). The study showed that compared with lovastatin model group, administration of daidzein (100 and 150 mg/kg/day) improved the weight loss of mice (Fig. 3A). Compared with lovastatin model group, the mice gastrocnemius muscle mass increased significantly (Fig. 3B). At the same time, we also found that after treatment with daidzein, the mice muscle grip strength was significantly restored (Fig. 3C), reflecting that the muscle function of mice was significantly improved after treatment with daidzein. Similarly, by H&E staining of mice gastrocnemius muscle, we could see that administration of daidzein (100 and 150 mg/kg/day) improved lovastatin-induced muscle fiber atrophy in mice (Fig. 3D). The mice serum creatine kinase, which generally used to test skeletal muscle damage, was significantly increased after lovastatin administration, but significantly decreased after administration of daidzein (100 and 150 mg/kg/day, Fig. 3E). As shown in (Fig. 3F), the up-regulation of two muscle degradation proteins Atrogin-1 and MuRF-1 induced by lovastatin in mice gastrocnemius was also reversed by daidzein. Taken together, these results indicated that daidzein could improve lovastatin-induced skeletal muscle atrophy in mice.

### Knockdown of FOXO3a reverses lovastatin-induced C2C12 myotubes atrophy

**Fig. 4:**
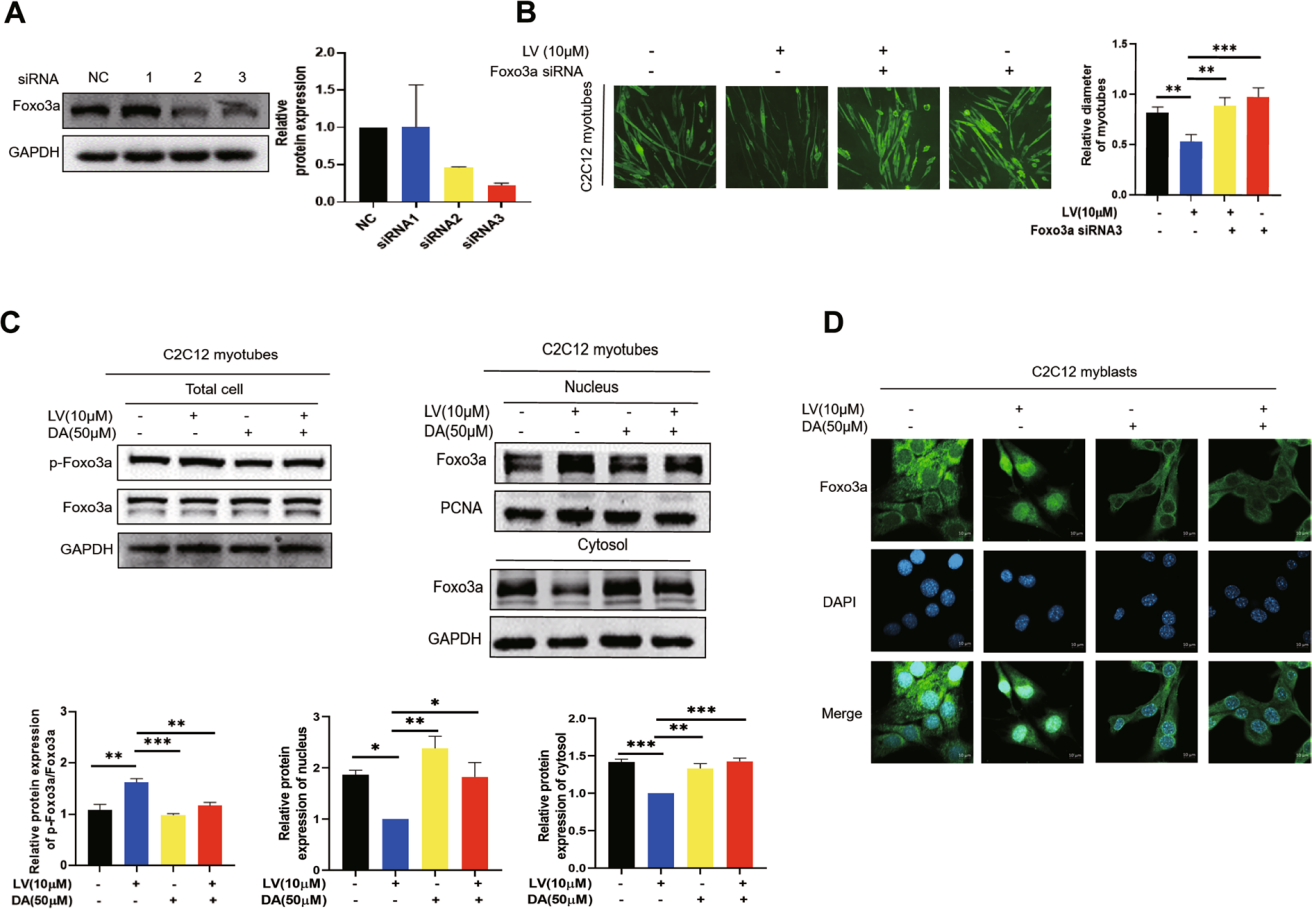
Knockdown of FOXO3a reverses lovastatin-induced C2C12 myotubes atrophy. **A** C2C12 myotubes were administered with three different FOXO3a siRNA (100 nM) for 48 h, proteins were extracted and immunoblotting was used to test the knockdown efficiency. **B** C2C12 myotubes were administered with FOXO3a siRNA for 48 h and incubated with lovastatin for another 24 h, MyHC was stained, and the inverted microscope was used to capture the pictures; C2C12 myotubes were calculated and analyzed with ImageJ. **C** C2C12 myotubes were administered with 10 µmol/L lovastatin (LV) + 50 µmol/L daidzein (DA) for 24 h, nuclear and cytoplasmic proteins were extracted. FOXO3a in total cell, nucleus and cytosol were tested by immunoblotting. The image J was used to quantify the protein. **D** C2C12 cells were administered with lovastatin (LV) + daidzein (DA) for 24 h, FOXO3a and DAPI were stained, and the confocal microscopy were used to capture the images. The mean ± SEM was used to represent the data. *p < 0.05; **p < 0.01; ***p < 0.001, (n = 3)

The above results proved that daidzein could improve lovastatin-induced skeletal muscle atrophy. However, the specific mechanism remains unclear. The transcription of Atrogin-1 and MuRF-1 is regulated by the FOXO family of forkhead protein transcription factors. FOXO proteins mainly include 4 subtypes: FOXO1, FOXO3, FOXO4 and FOXO6 [41]. Study showes that metformin induces muscle atrophy through HDAC6 and FOXO3a transcriptional regulation of myostatin [42]. Lovastatin inhibites cardiac hypertrophy by regulating p21 through regulating FOXO3a [17]. All these indicate that FOXO3a plays an important role in muscle diseases, whether FOXO3a is involved in lovastatin-induced skeletal muscle atrophy needed further study. Firstly, we used three small interfering RNA to knockdown FOXO3a and found that siRNA3 had the best knockdown efficiency of FOXO3a. (Fig. 4A) Therefore we chose siRNA3 for experiment and found that knockdown of FOXO3a reversed lovastatin-induced C2C12 myotubes atrophy (Fig. 4B), indicating that FOXO3a played an important function in lovastatin-induced C2C12 myotubes atrophy. Studies show that statins can increase the phosphorylation of FOXO3a to promote FOXO3a to enter the nucleus in skeletal muscle cells [12]. The results showed that lovastatin increased the phosphorylation of FOXO3a and promoted the nuclear transfer of FOXO3a in C2C12 myotubes, but administration of daidzein down-regulated the p-FOXO3a and reversed the nuclear transfer of FOXO3a (Fig. 4C). The immunofluorescence figures also reflected that daidzein could reverse lovastatin-induced nuclear transfer of FOXO3a (Fig. 4D). Besides, in lovastatin induced-zebrafish model, lovastatin promoted the nuclear transfer of FOXO3a. However, daidzein also reversed the nuclear transfer of FOXO3a compared with LV group in zebrafish (Fig. S4 D). The above results showed that knockdown of FOXO3a reversed lovastatin-induced C2C12 myotubes atrophy, and administration of daidzein down-regulated the p-FOXO3a inhibited the nuclear transfer of FOXO3a caused by lovastatin and reduced *FbxoO32* and *Murf-1* mRNA levels, thus reversing lovastatin-induced C2C12 myotubes atrophy.

### Daidzein reverses lovastatin-induced C2C12 myotubes atrophy depending on AMPK/FOXO3a pathway

**Fig. 5:**
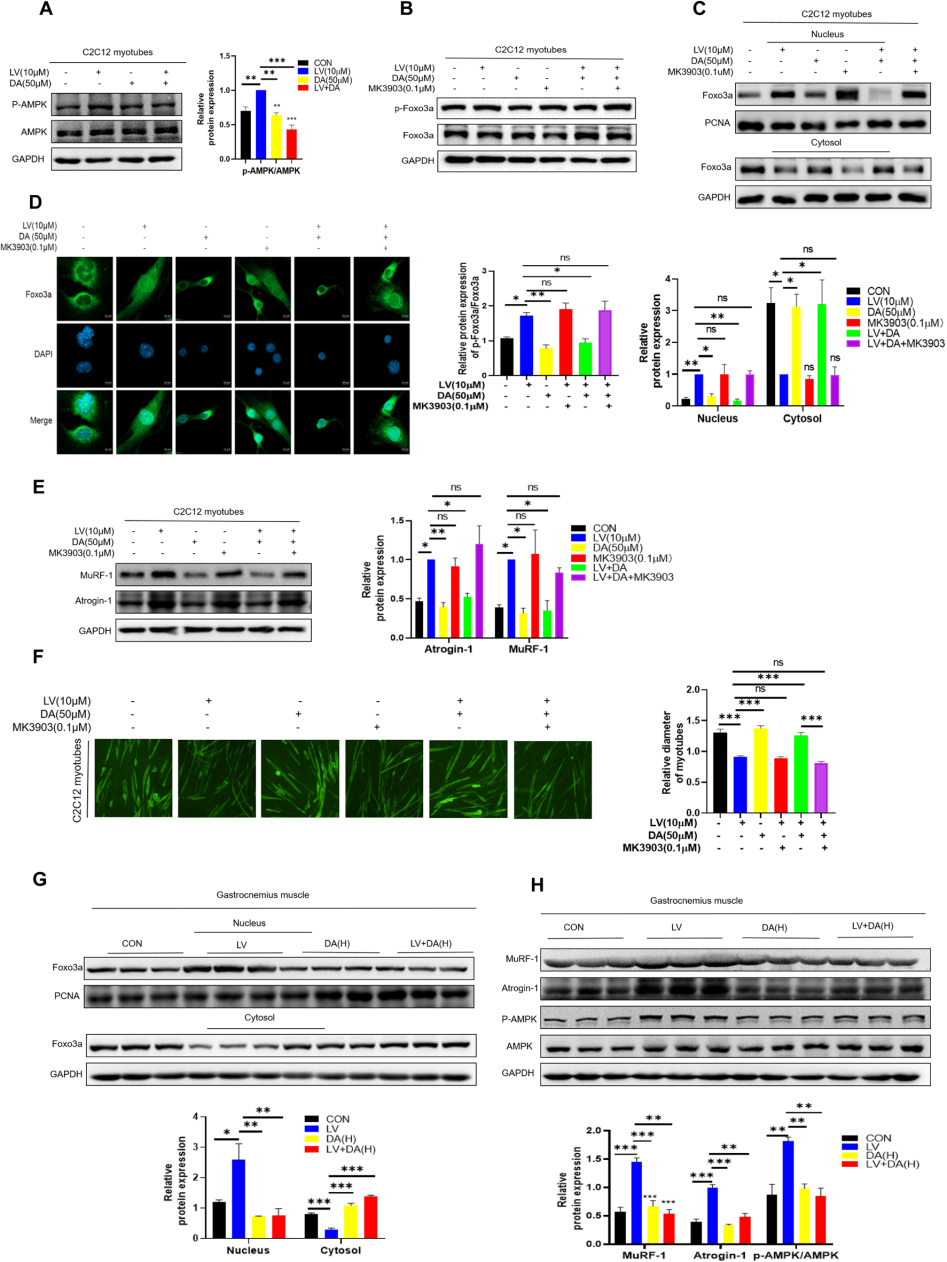
Daidzein reverses lovastatin-induced C2C12 myotubes atrophy depending on AMPK/FOXO3a pathway.** A** C2C12 myotubes were administered with 10 µmol/L lovastatin + 50 µmol/L daidzein for 24 h, cell proteins were extracted, immunoblotting was used to test the AMPK and p-AMPK expression level(n = 3). **B** C2C12 myotubes were administered with lovastatin, daidzein, 0.1 µmol/L MK-3903, lovastatin + daidzein and lovastatin + daidzein + 0.1 µmol/L MK-3903 for 24 h, cell proteins were extracted, immunoblotting was used to test the p-FOXO3a and FOXO3a expression level. **C** C2C12 myotubes were respectively administered with lovastatin, daidzein, MK-3903, lovastatin + daidzein and lovastatin + daidzein + MK-3903 for 24 h, the nuclear and cytoplasmic proteins were extracted. Immunoblotting was used to test the FOXO3a expression in cytoplasmic and nucleus. The image J was used to quantify the protein. **D** C2C12 cells were respectively treated with lovastatin, daidzein, MK-3903, lovastatin + daidzein and lovastatin + daidzein + MK-3903 for 24 h, FOXO3a and DAPI were stained, and the confocal microscopy was used to capture the images. **E** C2C12 myotubes were were treated as in Figure B, cell proteins were extracted, and immunoblotting was used to test the proteins expression. The image J was used to quantify the protein (n = 3). **F** C2C12 myotubes were treated as in Figure B and then MyHC was stained, and using the inverted microscope to capture the pictures; C2C12 myotubes were calculated and analyzed with ImageJ. **G** Nuclear and cytoplasmic protein from the gastrocnemius muscle of mice were collected in each group and immunoblotting was used to test the proteins expression. The image J was used to quantify the protein. **H** Extract protein from gastrocnemius muscle of mice and immunoblotting was used to test the protein expression levels. The image J was used to quantify the protein. The mean ± SEM was used to represent the data. *p < 0.05; **p < 0.01; ***p < 0.001, (n = 3)

AMPK regulates bioenergy metabolism and a variety of growth-related pathways, including JAK [43], p53 [44] and FOXO3a [45]. As an upstream signal, AMPK phosphorylates FOXO3a at 6 sites, promoting FOXO3a to enter the nucleus, thus causing biological effects [45]. Studies have reported that atorvastatin attenuates cardiac hypertrophy by activating AMPK [46]. Administered with long-term and short-term statin therapy increased the AMPKα activity in human skeletal muscle [47]. Daidzein attenuated skeletal muscle atrophy induced by chemotherapy drug in mice by inhibiting AMPK activation [36]. Therefore, AMPK and FOXO3a were closely related to muscle atrophy. In our study, we found that daidzein inhibited lovastatin-induced AMPK activation in C2C12 myotubes (Fig. 5A). The MK-3903 (an AMPK-selective agonist) increased the p-FOXO3a and reversed the effects of daidzein on the level of p-FOXO3a in lovastatin-induced C2C12 myotubes (Fig. 5B). The results (Fig. 5C) indicated that the effect of daidzein on nuclear transfer of FOXO3a was reversed when MK-3903 was added at the same time. Meanwhile, the immunofluorescence figures also reflected that MK-3903 reversed the effect of daidzein on reversing the lovastatin-induced nuclear transfer of FOXO3a (Fig. 5D). Besides, addition of MK-3903 reversed the action of daidzein on Atrogin-1 and MuRF-1 in lovastatin-induced C2C12 myotubes (Fig. 5E). Immunofluorescence images also showed that MK-3903 reversed the alleviation of lovastatin-induced C2C12 myotubes atrophy by daidzein (Fig. 5F). Similarly, in lovastatin-induced mouse muscle atrophy model, we performed nucleocytoplasmic separation of mouse gastrocnemius muscle tissue and detected the expression of FOXO3a. It was discovered that the nuclear transfer of FOXO3a increased in lovastatin model group, but when treated with daidzein, the nuclear transfer of FOXO3a decreased compared with CON (Fig. 5G). Compared with CON group, we also found that the level of p-AMPK was increased in lovastatin model group, but when treated with daidzein, the level p-AMPK was down-regulated (Fig. 5H). Meanwhile, we conducted molecular docking and found that daidzein could bind to the amino acids of the AMPK α1 subunit which suggested that daidzein might influence AMPK activation through its binding(Fig. S5 A). These results indicated that daidzein reversed lovastatin-induced C2C12 myotubes atrophy depending on AMPK/FOXO3a pathway.

### Pharmacological inhibition of AMPK reverses lovastatin-induced C2C12 myotubes atrophy

**Fig. 6:**
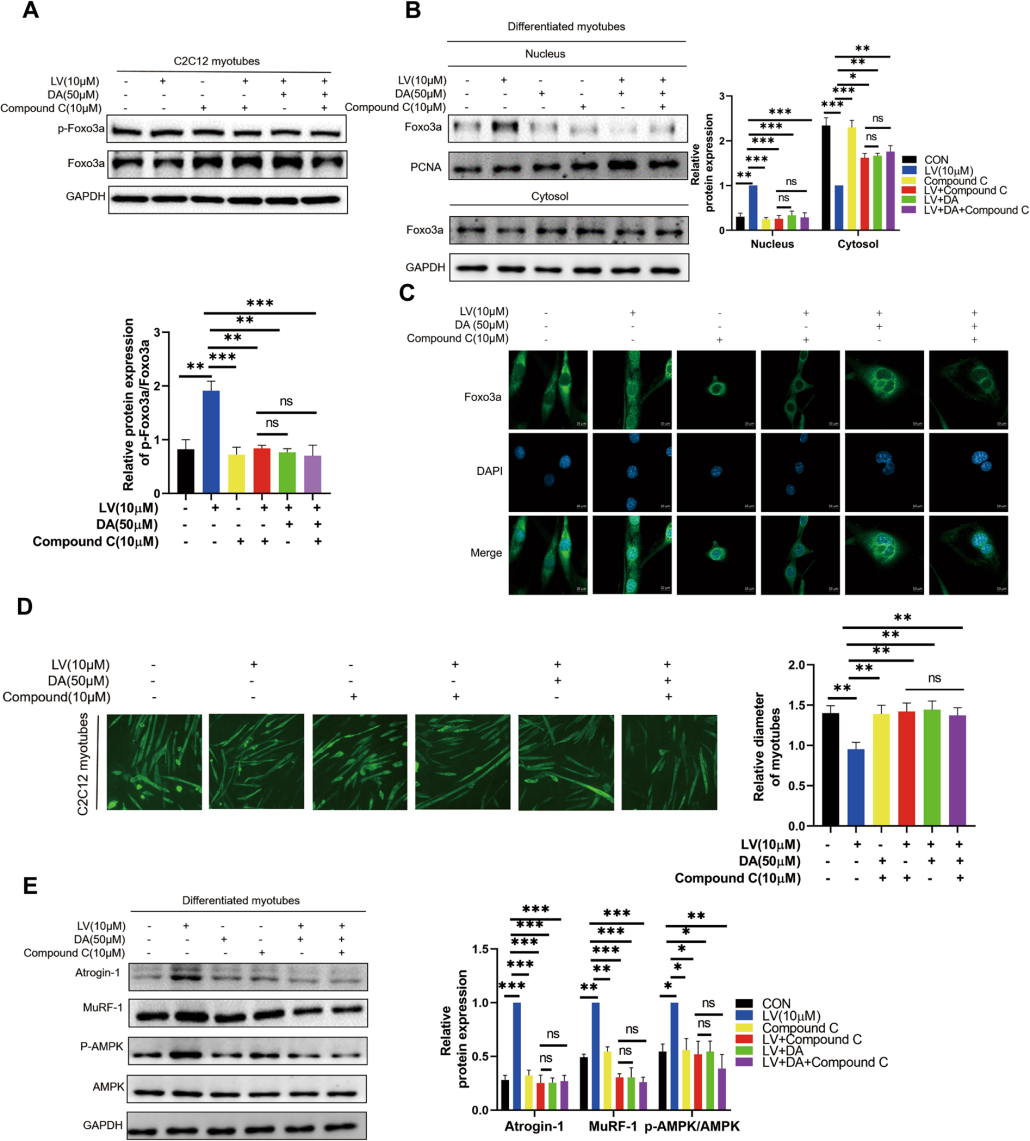
Pharmacological inhibition of AMPK reverses lovastatin-induced C2C12 myotubes atrophy.** A** C2C12 myotubes were respectively administered with 10 µmol/L lovastatin, 50 µmol/L daidzein, 10 µmol/L Compound C, 10 µmol/L lovastatin + 50 µmol/L daidzein and 10 µmol/L lovastatin + 50 µmol/L daidzein + 10 µmol/L Compound C for 24 h, cell proteins were extracted, immunoblotting was used to test the p-FOXO3a and FOXO3a expression level. **B** C2C12 myotubes were respectively administered with lovastatin, daidzein, Compound C, lovastatin + daidzein and lovastatin + daidzein + Compound C for 24 h, nuclear and cytoplasmic proteins were extracted. FOXO3a in nucleus and cytosol were tested by immunoblotting. The image J was used to quantify the protein. **C** C2C12 cells were respectively treated with lovastatin, daidzein, Compound C, lovastatin + daidzein and lovastatin + daidzein + Compound C for 24 h, FOXO3a and DAPI were stained, and the confocal microscopy was used to capture the images. **D** C2C12 myotubes were respectively administered with lovastatin, daidzein, Compound C, lovastatin + daidzein and lovastatin + daidzein + Compound C for 24 h, MyHC was stained, and the inverted microscope was used to capture the pictures; C2C12 myotubes were calculated and analyzed with ImageJ**. E** C2C12 myotubes were respectively administered with lovastatin, daidzein, Compound C, lovastatin + daidzein and lovastatin + daidzein + Compound C for 24 h, the total proteins were extracted, and immunoblotting was used to test the proteins level. The image J was used to quantify the protein. The mean ± SEM was used to present the data. *p < 0.05; **p < 0.01; ***p < 0.001, (n = 3)

The above results illustrated that daidzein reversed lovastatin-induced C2C12 myotubes atrophy by inhibiting AMPK activation. However, whether direct inhibition of AMPK activity can reverse lovastatin-induced C2C12 myotubes atrophy remains undetermined. We found that adding Compound C or daidzein alone and their combination had the same effect on reversing the effect of lovastatin on the p-FOXO3a expression and the nuclear translocation of FOXO3a in lovastatin-induced C2C12 myotubes (Fig. 6A, B). Meanwhile, the immunofluorescence figures also reflected that adding Compound C or daidzein alone and their combination had the same effect on reversing the lovastatin-induced nuclear transfer of FOXO3a (Fig. 6C). Similarly, when daidzein and Compound C were added in combination, the muscle-protective effect was not potentiated compared to that by using Compound or daidzein alone (Fig. 6D). Besides, addition of Compound C or daidzein alone and their combination had the same effect on reversed the protective effects of daidzein in lovastatin-induced C2C12 myotubes (Fig. 6E). AMPK knock-down C2C12 myotubes was also constructed to validate our conclusion. In our study, knockdown of AMPK inhibited the activity of AMPK and downregulated the expression level of p-AMPK. We found that knockdown of AMPK or daidzein alone and their combination had the same effect on reversing the effect of lovastatin on the p-FOXO3a expression and the nuclear translocation of FOXO3a in lovastatin-induced C2C12 myotubes. Meanwhile, knockdown of AMPK also downregulated the expression of Atrogin-1 and MuRF-1 which was equivalent to daidzein alone and their combination (Fig. S3A-D). The above results indicated that daidzein presented anti-atrophy effect through blocking AMPK activation and then decreased the p-FOXO3a expression.

### Daidzein improves muscle atrophy in mice caused by lovastatin by inhibiting AMPK activation

**Fig. 7:**
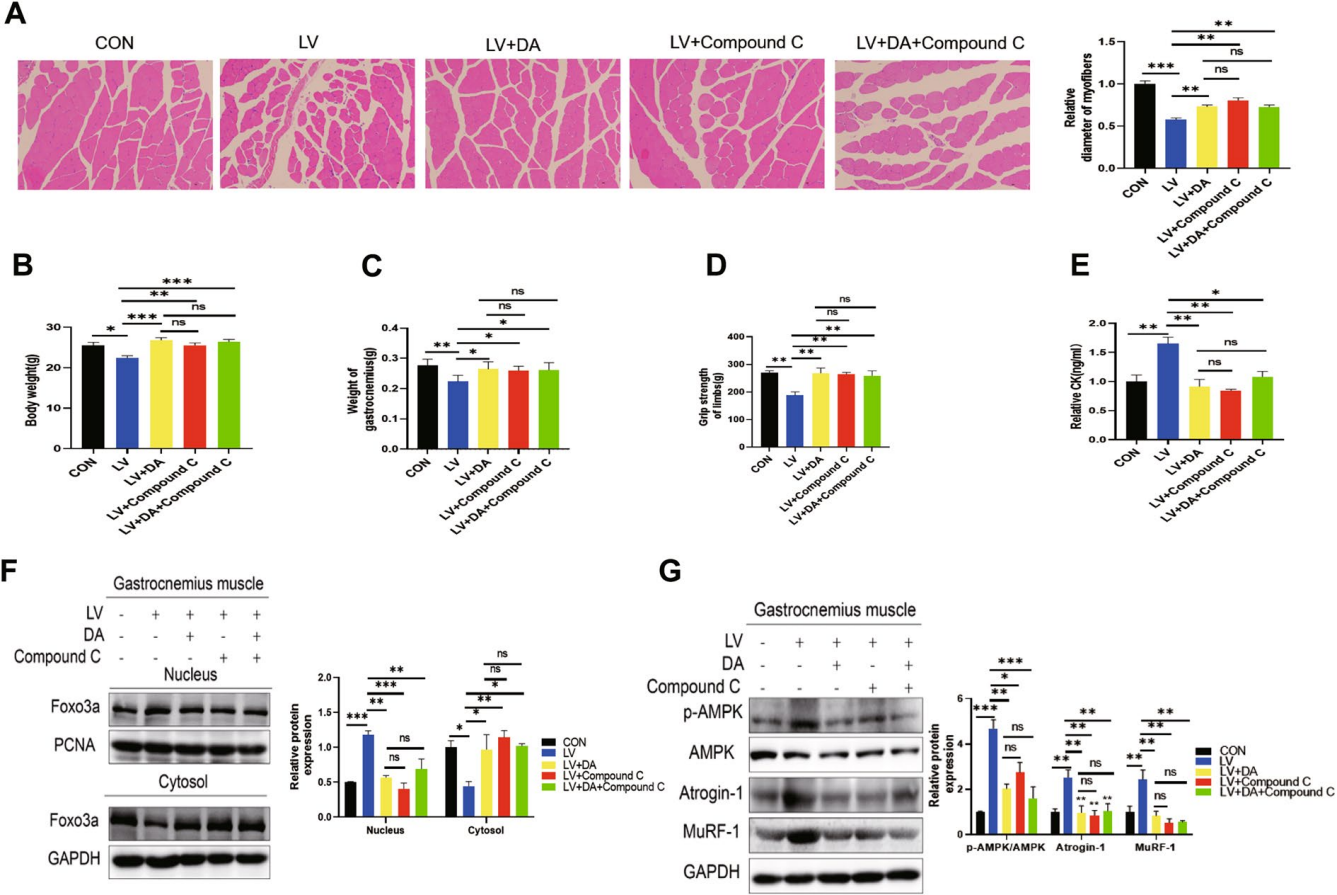
Daidzein improves muscle atrophy in mice caused by lovastatin by inhibiting AMPK activation. The mice were evenly divided into 5 groups. After treatment with LV (lovastatin, 80 mg/kg/day), DA (daidzein, 150 mg/kg/day), Compound C (20 mg/kg/day), (80 mg/kg/day) lovastatin + (150 mg/kg/day) daidzein, (80 mg/kg/day) lovastatin + (150 mg/kg/day) daidzein + (20 mg/kg/day) Compound C for 2 months and the mice were analyzed. **A** Hematoxylin–eosin staining of mice gastrocnemius muscle (scale bar = 50 µm). **B** Body weight. **C** Mass of gastrocnemius muscle. **D** Grip strength. **E** Serum CK activity. **F** Nuclear and cytoplasmic protein from the gastrocnemius muscle of mice were collected in each group, and immunoblotting was used to test the proteins expression. The image J was used to quantify the protein. **G** Extract protein from gastrocnemius muscle of mice in each group and immunoblotting was used to test the protein levels. The image J was used to quantify the protein (n = 3). The mean ± SEM was used to present the data. *p < 0.05; **p < 0.01; ***p < 0.001, (n = 3)

Above results showed that daidzein reversed lovastatin-induced C2C12 myotubes atrophy by inhibiting AMPK activation, thereby inhibiting the nuclear transfer of FOXO3a. In order to further confirm that daidzein could improve muscle atrophy in mice caused by lovastatin by inhibiting AMPK activation, we treated mice with Compound C and found that compared with lovastatin alone, Compound C improved the body weight, gastrocnemius muscle mass loss and muscle grip of mice (Fig. 7A–D). The muscle damage marker creatine kinase was also down-regulated after Compound C (Fig. 7E). Meanwhile, the expression of muscle degradation proteins MuRF-1, Atrogin-1 and the nuclear transfer of FOXO3a in gastrocnemius muscle of mice were all inhibited after administration of Compound C alone compared with the lovastatin model group (Fig. 7F, G). Furthermore, we found that the change of these indicators in the combined daidzein and Compound C group were consistent with those given alone in the Compound C group or the daidzein group, which showed that daidzein anti-atrophy effect may through blocking AMPK activation. The above results indicated that daidzein improved muscle atrophy in mice caused by lovastatin by inhibiting AMPK activation, thereby inhibiting the nuclear translocation of FOXO3a.

## Discussion

Statins-induced myopathy is the major side reaction of its long-term application. Currently, there are no good preventive methods and drugs to alleviate statin-induced muscle toxicity. Study shows that treatment with vitamin D improve muscle toxicity in mice caused by simvastatin by restoring mitochondrial cristae shape [48]. Insulin-like growth factor prevent statin-induced myotoxicity [12]. We previously find that statins can lower GGPP contents, resulting in statin-induced myotoxicity. [11]. Meanwhile, study indicates that exogenous supplementation of GGPP can improve statin-induced skeletal muscle atrophy, but dietary GGPP levels interferes with the effect of statins in treatment of experimental pulmonary arterial hypertension [49], indicating that supplementation of GGPP may affect the efficacy of statins. Therefore, it is necessary to find drugs that can prevent or treat statin-induced myotoxicity.

In this study, we demonstrated that daidzein could dose-dependently alleviate lovastatin-induced mice muscle atrophy, improve mouse gastrocnemius muscle quality, decrease creatine kinase levels and enhance mouse muscle grip strength, thus ameliorating skeletal muscle atrophy. Meanwhile, we found that daidzein reversed C2C12 myotubes atrophy caused by lovastatin. We also found that daidzein improved lovastatin-induced zebrafish muscle fibers damage and improved zebrafish's locomotor ability. Besides, daidzein has many different pharmacological activities such as lowering blood lipids and lowering blood sugar, which indicates that supplementation of daidzein may not affect the efficacy of statins. However, more animal models are needed to further determine whether daidzein can affect the efficacy of statin in the future.

Atrogin-1 and MuRF-1 are found to act crucial roles in muscle atrophy through gene gain and loss methods [7, 10]. Meanwhile, Atrogin-1 and MuRF-1 also act a key function in statin-induced skeletal muscle atrophy [13]. Soy isoflavones inhibit the expression of MuRF-1 through the activation of SIRT1 to inhibit muscle atrophy [50]. Supplementing a certain amount of isoflavones in diet can improve the muscle atrophy caused by tumor load in mice [37]. In our study, we found that daidzein inhibited the up-regulation of AMPK and FOXO3a phosphorylation caused by lovastatin, inhibited the nuclear transfer of the transcription factor FOXO3a, and down-regulated the transcription of *Fbxo32* and *Murf-1*. Study has found that spermidine can enhance autophagy and reduce apoptosis by regulating AMPK/FOXO3a axis when combines with exercise, thereby rescuing skeletal muscle atrophy in aging rats caused by D-gal [51]. Monotropein can improve muscle atrophy induced by dexamethasone by controlling the AKT/FOXO3a axis [52]. Meanwhile, knock-down FOXO3a reversed lovastatin-induced C2C12 myotubes atrophy, which further supports that FOXO3a plays a key function in statin-induced skeletal muscle atrophy. The main mechanism of regulating FOXO3a is subcellular localization, including phosphorylation, acetylation and ubiquitination through post-transcriptional regulation. Different upstream signals have different effects on the localization of FOXO cells. AMPK, as an upstream signal, phosphorylates FOXO3a at six sites. Statins can activate AMPK mainly through two mechanisms. On the one hand, AMPK, as an energy receptor, can sense the changes of intracellular cholesterol and statins activate AMPK by reducing the content of intracellular cholesterol. On the other hand, statins increase the phosphorylation of AMPK at threonine 172 by enhancing Rac1 activation, thereby activating AMPK [27]. Our study found that lovastatin induced skeletal muscle atrophy by activating AMPK. And daidzein or knockdown of AMPK could improve lovastatin-induced muscle atrophy through blockage of abnormal activation of AMPK. Besides, an AMPK agonist Metformin is a classic hypoglycemic drug by inhibiting glucose absorption, but its long-term application is continuously challenged by potential skeletal muscle atrophy [20–22, 42]. These results indicate that activation of AMPK may cause skeletal muscle atrophy.

In our study, we found that knockdown of FOXO3a reversed lovastatin-induced skeletal muscle atrophy (Fig. 4B), indicating that FOXO3a played an important function in lovastatin-induced skeletal muscle atrophy. The results showed that lovastatin increased the phosphorylation of FOXO3a and promoted the nuclear transfer of FOXO3a in C2C12 myotubes (Fig. 4C). However, how lovastatin increases the phosphorylation of FOXO3a needs to further study. In our study, we found that adding Compound C (an-AMPK selective inhibitor) reversed the effect of lovastatin on the p-FOXO3a expression and the nuclear translocation of FOXO3a in lovastatin-induced C2C12 myotubes (Fig. 6A, B). Compound C reversed lovastatin-induced C2C12 myotubes atrophy (Fig. 6D, E). Besides, we found that knockdown of AMPK also reversed lovastatin-induced FOXO3a nuclear translocation to restrain the expression of muscle-related proteins Atrogin-1 and MuRF-1. (Fig. S3A–D). Above all, we think that AMPK/FOXO3a plays an important role in statin-induced muscle atrophy.

Besides, we found that daidzein inactivated AMPK by inhibiting AMPK phosphorylation, thereby decreasing the expression of FOXO3a phosphorylation, further inhibiting the nuclear transfer of FOXO3a and alleviating the muscle atrophy induced by lovastatin. In C2C12 myotubes, administration of AMPK-selective inhibitor Compound C recapitulated the therapeutic effects of daidzein against lovastatin-induced myotubes atrophy, while the anti-atrophy effects of daidzein were lost in the presence of AMPK-selective agonist MK-3903. We found that the simultaneous administration of Compound C and daidzein was equivalent to the administration of Compound C or daidzein alone in alleviating lovastatin-induced muscle atrophy. It further illustrated that daidzein alleviated lovastatin-induced muscle atrophy possibly by inhibiting AMPK activation.

Although we find that daidzein can improve lovastatin-induced muscle atrophy through blockage of abnormal activation of AMPK/FOXO3a axis, we have not clearly explained the exact mechanism of AMPK in lovastatin-induced muscle atrophy. Meanwhile, how daidzein inhibits AMPK activity needs further study. Our goal was to find drugs that could alleviate lovastatin-induced muscle atrophy and explore its possible mechanisms. The results showed that daidzein could alleviate muscle atrophy induced by lovastatin. Mechanistic studies found that daidzein inhibited the activation of AMPK caused by lovastatin and inhibited the nuclear transfer of FOXO3a, thereby inhibiting the transcriptional expression of muscle degradation-related proteins. Our results also showed that daidzein could improve lovastatin-induced muscle grip in mice, but whether this was related to mitochondria needed further exploration. Besides, some studies have shown that statins can cause mitochondrial damage resulting in muscle toxicity. AMPK inhibition also causes a series downstream effects, such as autophagy related mechanisms [53], mitochondrial dysfunction [54], and so on. However, it remains to be investigated whether daidzein can alleviate lovastatin-induced muscle toxicity through its effects on mitochondria or autophagy and further research is needed to explore this relationship. In addition, the target of daidzein reversing lovastatin-induced muscle atrophy needs to be further explored, too. In view of the fact that there is no effective prevention and treatment method to alleviate muscle atrophy induced by statins, our study suggested that daidzein combination with lovastatin may be a promising prevention strategy. Daidzein is present predominantly in the form of glucosides in various plants, including red clover (*Trifolium pratense*), alfalfa (*Medicago sativa*) and soybean (*Glycine max*) [55]. Therefore, our findings might provide insights into support the botanical medicine-based therapies potential in management of statin-induced muscle disorders.

## Conclusion

**Fig. 8:**
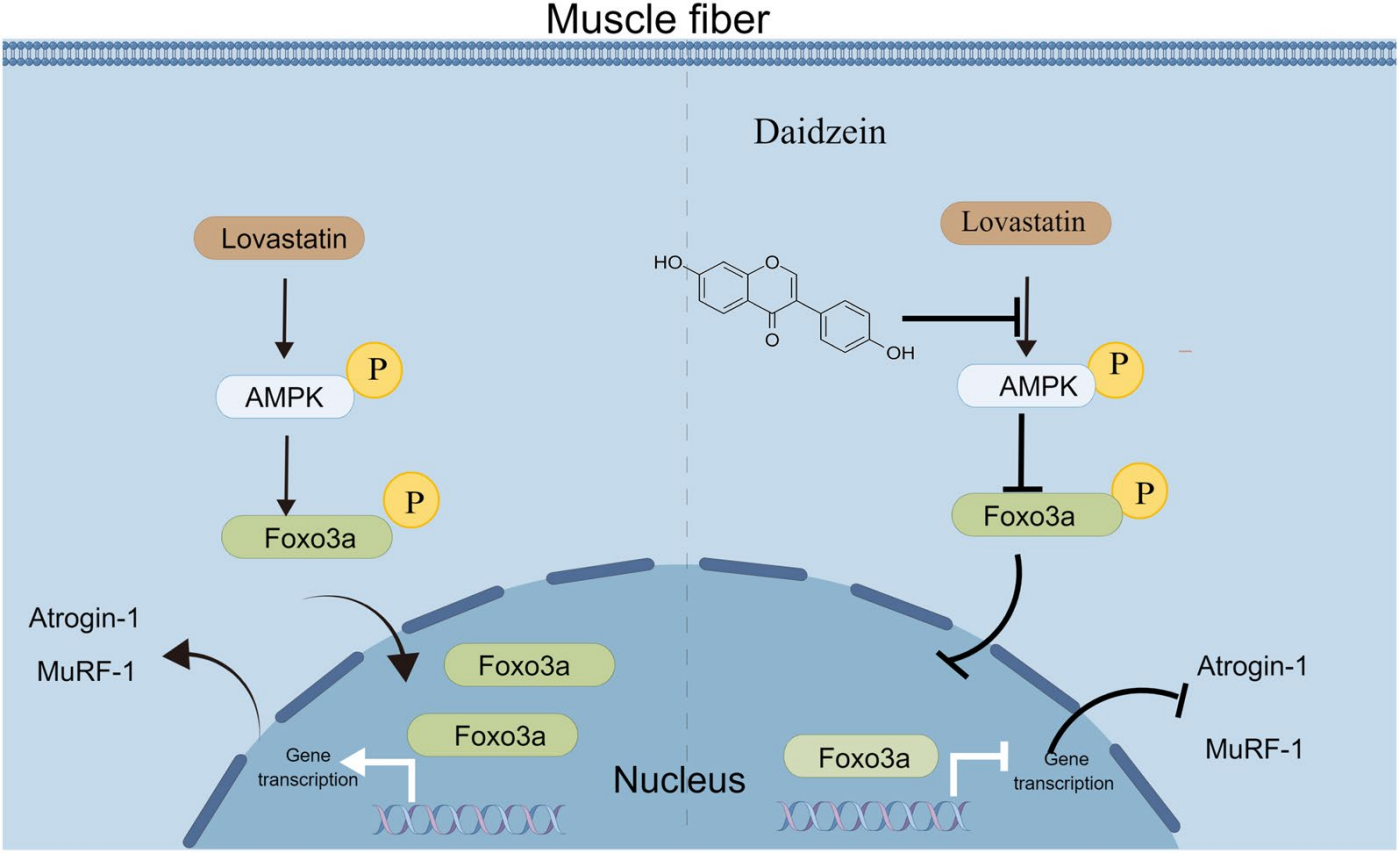
Mechanism diagram of daidzein improving lovastatin-induced skeletal muscle atrophy. Mechanistic studies revealed that statins induced the phosphorylation of FOXO3a by activating AMPK, leading to the nuclear translocation of FOXO3a and transcriptional activation of muscle protein degradation-related enzymes Atrogin-1 and MuRF-1, thereby initiating pathological muscle degradation processes. Our study identified daidzein as an effective component that ameliorates lovastatin-induced skeletal muscle atrophy through blockage of abnormal activation of AMPK/FOXO3a and transcriptional activation of genes encoding downstream muscle-related proteins

In this study, we demonstrated that daidzein could significantly improve mouse gastrocnemius muscle quality, decrease creatine kinase levels and enhance mouse muscle grip strength, thus ameliorating skeletal muscle atrophy. Mechanistic studies revealed that statins induced the phosphorylation of FOXO3a by activating AMPK, leading to the nuclear translocation of FOXO3a and transcriptional activation of muscle protein degradation-related enzymes Atrogin-1 and MuRF-1, thereby initiating pathological muscle degradation processes (Fig. 8). We found that daidzein effectively reverse the above process by restricting activation of AMPK/FOXO3a axis. Subsequent pharmacological intervention experiments showed that AMPK inhibitors could mimic the effects of daidzein, while AMPK agonists could significantly abolish the therapeutic effects of daidzein, further confirming our findings. Thus, our findings provide a potential phytomedicine-based therapy against lovastatin-induced skeletal muscle atrophy. And the potential anti-muscle atrophy effects and mechanisms of daidzein demonstrated in our study might provide insights into support the therapeutic potential by targeting AMPK/FOXO3a axis in the management of statin-induced muscle disorders.

## Supplementary Information

Supplementary material 1: Fig. S1. Daidzein reverses the lovastatin-induced zebrafish muscle fibers damage and improved zebrafish’s locomotor ability. A 48 h zebrafish embryos were divided into four groups. CON group was hatched with embryo culture medium 24 h. LV group was hatched with embryo medium containing lovastatin 24 h. LV+DA group was hatched with embryo medium containing lovastatin and daidzein 24 h. Then zebrafish juveniles were placed in a 48-well plate. Using zebrafish behavioral analysis system to detect the sports ability of zebrafish. B 48 h zebrafish embryos were divided into four groups. CON group was hatched with embryo culture medium 24 h. LV group was hatched with embryo medium containing lovastatin 24 h. LV+DA group was hatched with embryo medium containing lovastatin and daidzein 24 h. DA group was hatched with embryo culture medium containing daidzein 24 h. Then MyHC was stained, and the confocal microscopy was used to capture the images.

Supplementary material 2: Fig. S2. The effect of daidzein on the viability and intracellular ATP of C2C12 cells. A C2C12 cells were administered with 10 µmol/L lovastatin and 50 µmol/L daidzein for 24 h and intracellular ATP was tested with Microplate reader. B C2C12 cells were administered with different concentrations of daidzein for 24 h and incubated CCK-8 for 1.5 h, and tested with Microplate reader. The mean ± SEM was used to present the data. *p < 0.05; **p < 0.01; ***p < 0.001.

Supplementary material 3: Fig. S3. Knockdown of AMPK reverses lovastatin-induced C2C12 myotubes atrophy. A-C C2C12 myotubes were administered with AMPK siRNA for 48 h and incubated with lovastatin and daidzein for another 24 h, cellular protein and RNA were collected. Immunoblotting and RT-qPCR were used to test the expression level of Atrogin-1, MuRF-1, AMPK, p-AMPK, FOXO3a, p-FOXO3a. D C2C12 myotubes were administered with AMPK siRNA for 48 h and incubated with lovastatin and daidzein for another 24 h, nuclear and cytoplasmic proteins were extracted. FOXO3a in total cell, nucleus and cytosol were tested by immunoblotting.

Supplementary material 4: Fig. S4. Daidzein reverses the expression of the lovastatin-induced muscle-related protein Atrogin-1 and MuRF-1. A-C 48 h zebrafish embryos were divided into four groups. CON group was hatched with embryo culture medium 24 h. LV group was hatched with embryo medium containing lovastatin 24 h. LV+DA group was hatched with embryo medium containing lovastatin and daidzein 24 h, protein and RNA were collected. Immunoblotting and RT-qPCR were used to test the expression level of Atrogin-1, MuRF-1. D 48 h zebrafish embryos were divided into four groups. CON group was hatched with embryo culture medium 24 h. LV group was hatched with embryo medium containing lovastatin 24 h. LV+DA group was hatched with embryo medium containing lovastatin and daidzein 24 h, nuclear and cytoplasmic proteins were extracted. FOXO3a in total zebrafish, nucleus and cytosol were tested by immunoblotting.

Supplementary material 5: Fig. S5. Daidzein may influence AMPK activation through binding the amino acids of the AMPK α1. A docking analysis was used to predict the binding of daidzein to AMPK

## Data Availability

The data generated during this study are available if the requests are reasonable.
