# Supplementary figures and images for: Daidzein improves muscle atrophy caused by lovastatin by regulating the AMPK/FOXO3a axis
Source: Chin Med. 2024 Dec 31;19:180. doi: 10.1186/s13020-024-01034-5 (PMC11686997; doi:10.1186/s13020-024-01034-5)

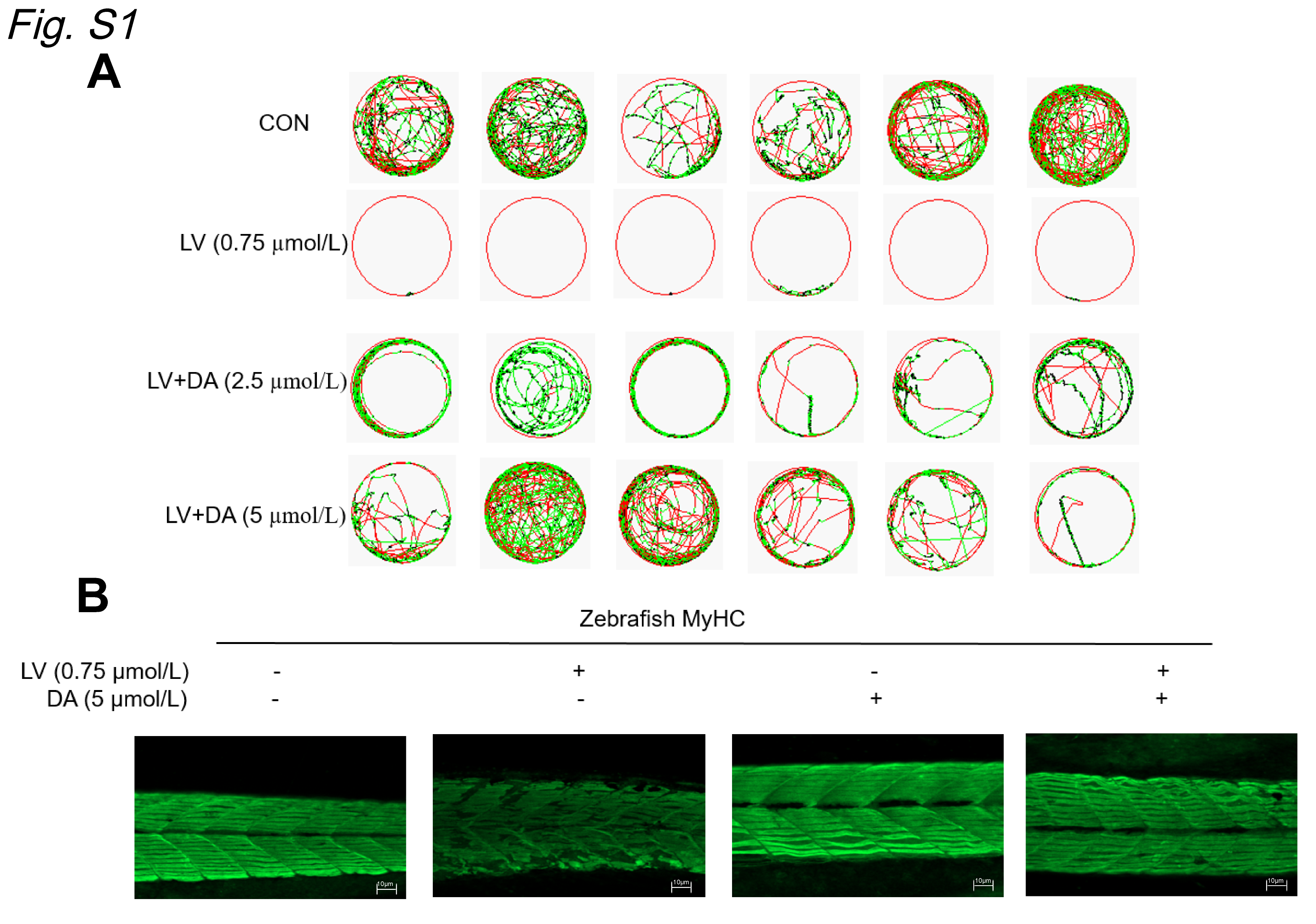

Supplement: Supplementary file 1 — Supplementary material 1: Fig. S1. Daidzein reverses the lovastatin-induced zebrafish muscle fibers damage and improved zebrafish’s locomotor ability. A 48 h zebrafish embryos were divided into four groups. CON group was hatched with embryo culture medium 24 h. LV group was hatched with embryo medium containing lovastatin 24 h. LV+DA group was hatched with embryo medium containing lovastatin and daidzein 24 h. Then zebrafish juveniles were placed in a 48-well plate. Using zebrafish behavioral analysis system to detect the sports ability of zebrafish. B 48 h zebrafish embryos were divided into four groups. CON group was hatched with embryo culture medium 24 h. LV group was hatched with embryo medium containing lovastatin 24 h. LV+DA group was hatched with embryo medium containing lovastatin and daidzein 24 h. DA group was hatched with embryo culture medium containing daidzein 24 h. Then MyHC was stained, and the confocal microscopy was used to capture the images. [file 13020_2024_1034_MOESM1_ESM.tif]

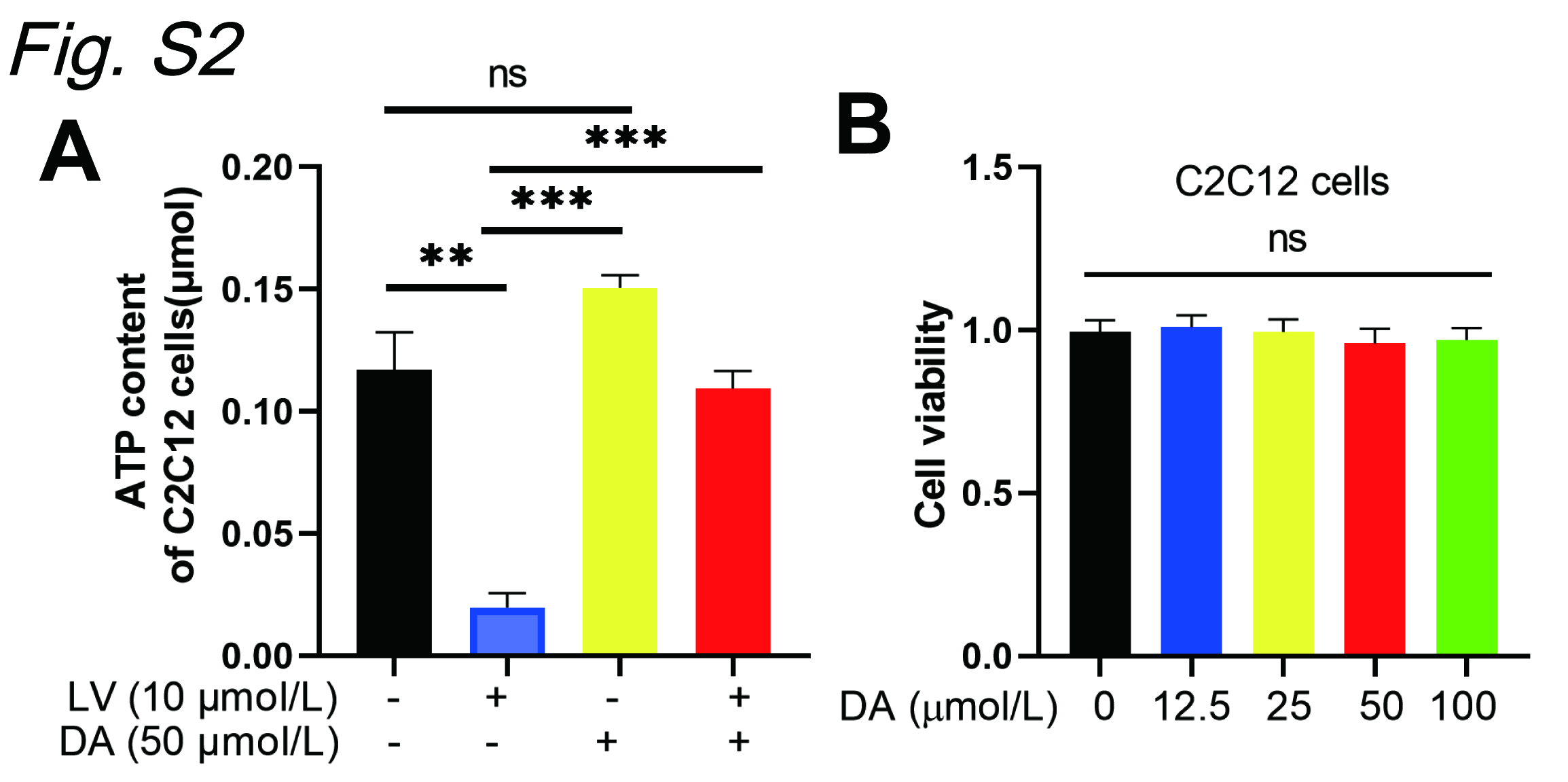

Supplement: Supplementary file 2 — Supplementary material 2: Fig. S2. The effect of daidzein on the viability and intracellular ATP of C2C12 cells. A C2C12 cells were administered with 10 µmol/L lovastatin and 50 µmol/L daidzein for 24 h and intracellular ATP was tested with Microplate reader. B C2C12 cells were administered with different concentrations of daidzein for 24 h and incubated CCK-8 for 1.5 h, and tested with Microplate reader. The mean ± SEM was used to present the data. *p < 0.05; **p < 0.01; ***p < 0.001. [file 13020_2024_1034_MOESM2_ESM.tif]

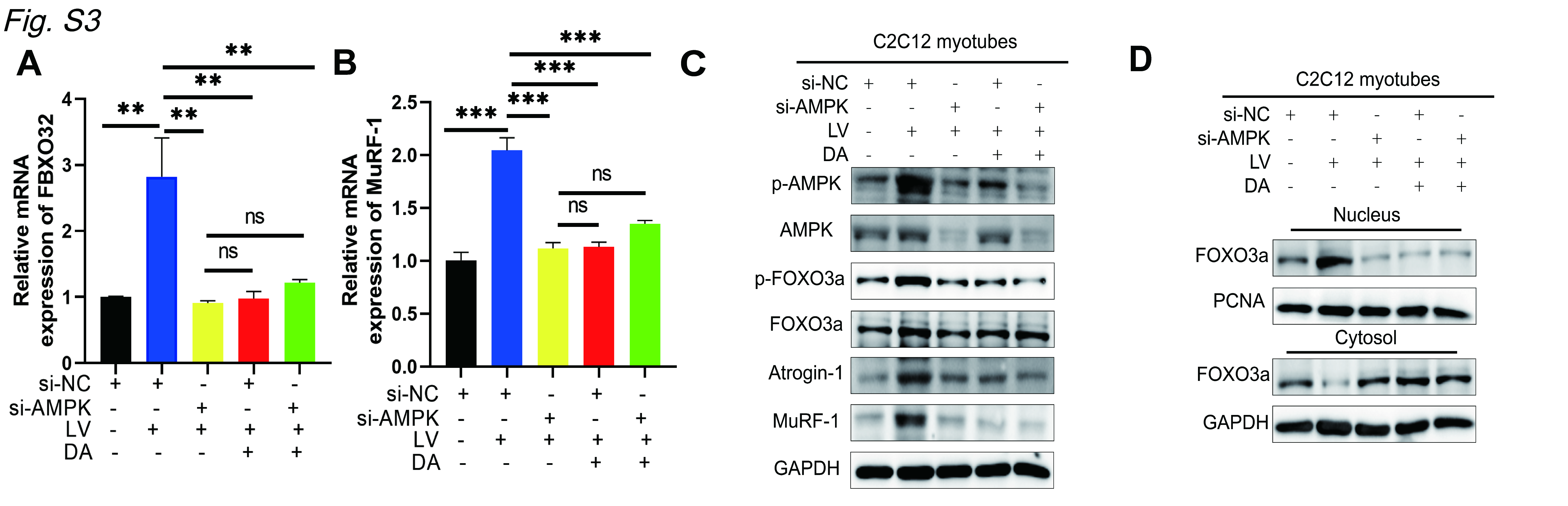

Supplement: Supplementary file 3 — Supplementary material 3: Fig. S3. Knockdown of AMPK reverses lovastatin-induced C2C12 myotubes atrophy. A-C C2C12 myotubes were administered with AMPK siRNA for 48 h and incubated with lovastatin and daidzein for another 24 h, cellular protein and RNA were collected. Immunoblotting and RT-qPCR were used to test the expression level of Atrogin-1, MuRF-1, AMPK, p-AMPK, FOXO3a, p-FOXO3a. D C2C12 myotubes were administered with AMPK siRNA for 48 h and incubated with lovastatin and daidzein for another 24 h, nuclear and cytoplasmic proteins were extracted. FOXO3a in total cell, nucleus and cytosol were tested by immunoblotting. [file 13020_2024_1034_MOESM3_ESM.tif]

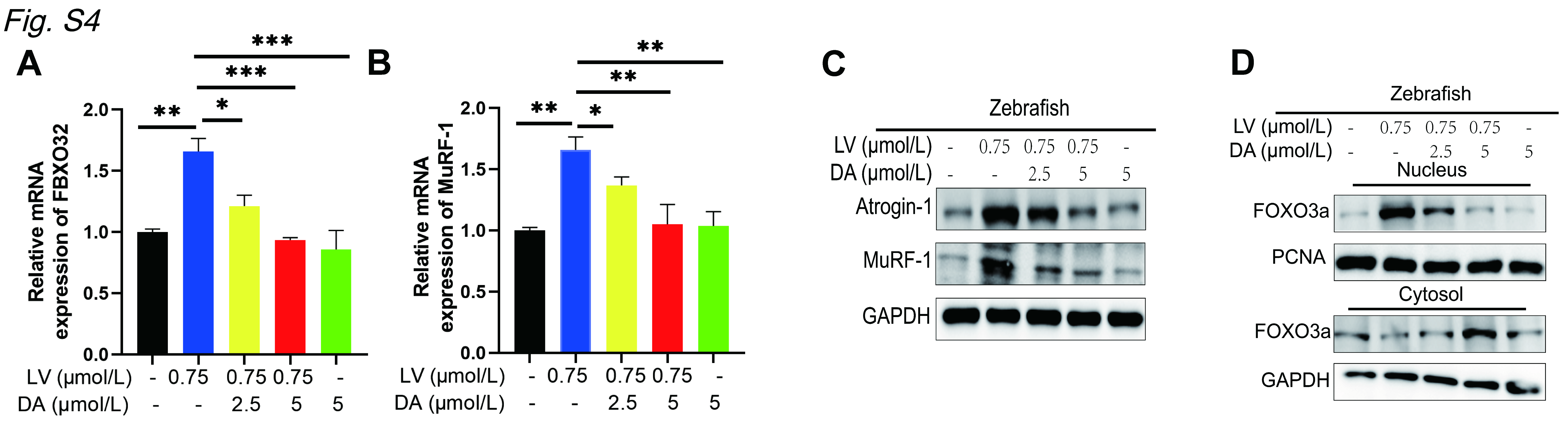

Supplement: Supplementary file 4 — Supplementary material 4: Fig. S4. Daidzein reverses the expression of the lovastatin-induced muscle-related protein Atrogin-1 and MuRF-1. A-C 48 h zebrafish embryos were divided into four groups. CON group was hatched with embryo culture medium 24 h. LV group was hatched with embryo medium containing lovastatin 24 h. LV+DA group was hatched with embryo medium containing lovastatin and daidzein 24 h, protein and RNA were collected. Immunoblotting and RT-qPCR were used to test the expression level of Atrogin-1, MuRF-1. D 48 h zebrafish embryos were divided into four groups. CON group was hatched with embryo culture medium 24 h. LV group was hatched with embryo medium containing lovastatin 24 h. LV+DA group was hatched with embryo medium containing lovastatin and daidzein 24 h, nuclear and cytoplasmic proteins were extracted. FOXO3a in total zebrafish, nucleus and cytosol were tested by immunoblotting. [file 13020_2024_1034_MOESM4_ESM.tif]

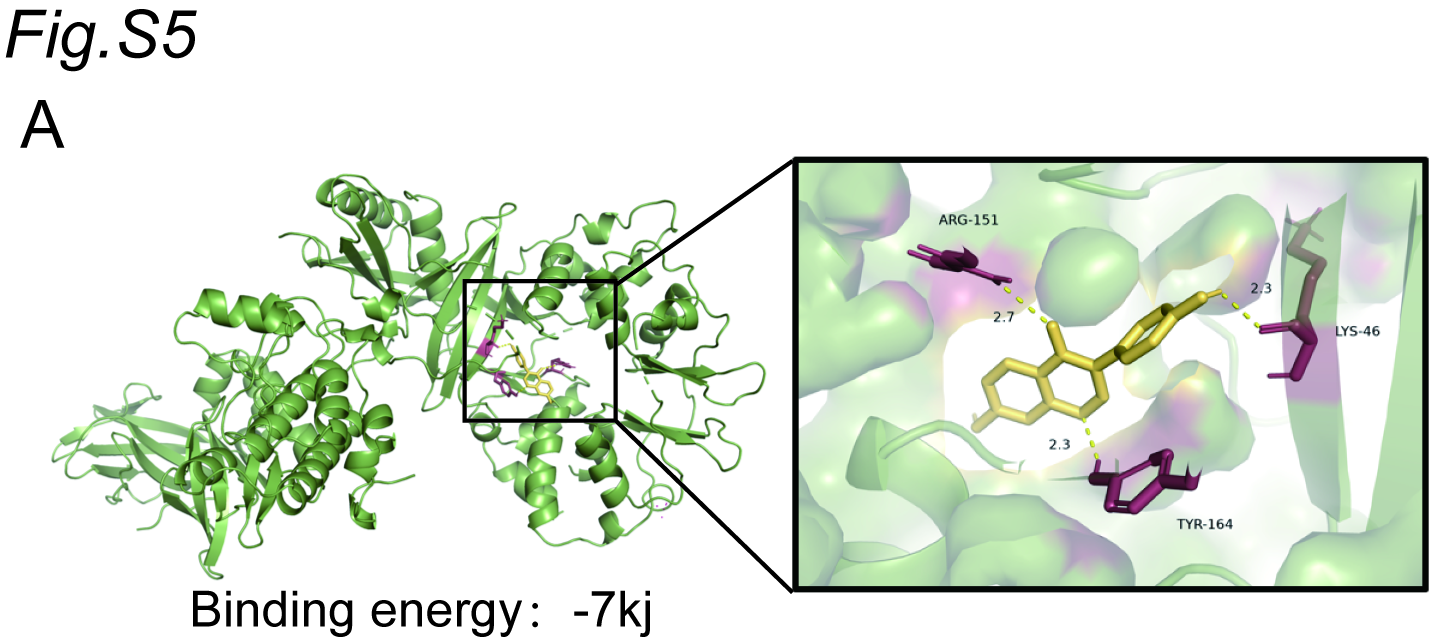

Supplement: Supplementary file 5 — Supplementary material 5: Fig. S5. Daidzein may influence AMPK activation through binding the amino acids of the AMPK α1. A docking analysis was used to predict the binding of daidzein to AMPK [file 13020_2024_1034_MOESM5_ESM.tif]
